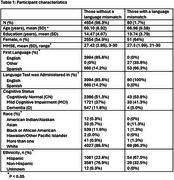# The Impact of Language Mismatch on Mini Mental State Examination Scores is Moderated by Years of Education

**DOI:** 10.1002/alz70857_101092

**Published:** 2025-12-24

**Authors:** Isabella Hoang, Joe Strong, Jacqueline Pontes Monteiro, Matt Glittenberg, Ozioma C. Okonkwo

**Affiliations:** ^1^ Wisconsin Alzheimer's Disease Research Center, School of Medicine and Public Health, University of Wisconsin‐Madison, Madison, WI, USA; ^2^ University of Kentucky, Lexington, KY, USA

## Abstract

**Background:**

In predominantly English‐speaking countries, those whose first language is not English may receive delayed cognitive diagnoses. This may be attributed to language barriers in cognitive testing such as the Mini‐Mental Status Examination (MMSE). There is a gap in the literature regarding the effects of administering the MMSE in a language other than one's first language and how factors such as education, sex, and cognitive diagnosis influence the results. This study uses the Alzheimer's Disease Neuroimaging Initiative (ADNI) and the Health and Aging Brain Study – Health Disparities (HABS‐HD) to examine this gap.

**Method:**

ADNI (*n* = 2202) and HABS‐HD (*n* = 2532) data were merged. MMSE scores showed a strong negative skewness, statistical analyses included Mann‐Whitney and Kruskal‐Wallis to compare MMSE scores across cognitively normal (CN), mild cognitive impairment (MCI) and dementia (D) groups, or between sex. Generalized Linear Model (GLM) with the link function log was used to examine the impact of language mismatches, defined by a difference in one's first language and the language the MMSE was administered in, and its interactions with education, cognitive diagnosis, age, and sex on MMSE scores.

**Result:**

80 participants have a language mismatch (ADNI = 32, HABS‐HD = 48) (Table 1). Years of education moderated the effect of language mismatch in the interaction term model (β = 0.007; *p* =  0.007); as education increases, the effect of language mismatch on MMSE score becomes less negative or more positive. No significant interaction effects were found between language mismatch and diagnosis (β = ‐0.012; *p* =  0.89), sex (β = ‐0.015; *p* =  0.46), or age (β = 0.001; *p* =  0.63).

**Conclusion:**

Years of education significantly moderates the association between language mismatch and MMSE score. This study adds nuance to considering education and language mismatches when using MMSE in modeling cognitive outcomes.